# Evaluation of cell viability and metabolic activity of a 3D cultured human epidermal model using a dynamic autoradiographic technique with a PET radiopharmaceutical

**DOI:** 10.1038/s41598-019-47153-0

**Published:** 2019-07-23

**Authors:** Toru Sasaki, Junya Tamaki, Kentaro Nishizawa, Takahiro Kojima, Ryoich Tanaka, Ryotaro Moriya, Haruyo Sasaki, Hiroko Maruyama

**Affiliations:** 1 Department of Medical Engineering and Technology, Kitasato University School of Allied of Health Sciences, 1-15-1 Kitasato, Sagamihara, Kanagawa 252-0373 Japan; 2 Research Facility of Regenerative Medicine and Cell Design, Kitasato University School of Allied of Health Sciences, 1-15-1 Kitasato, Sagamihara, Kanagawa 252-0373 Japan

**Keywords:** Tissue engineering, Molecular imaging

## Abstract

Quality control of tissues and organs for transplant is important to confirm their safety and effectiveness for regenerative medicine. However, quality evaluation is only carried out using a limited range of inspection criteria, because many of the available evaluation tests are invasive. In order to explore the potential of 2-[^18^F]fluoro-2-deoxy-D-glucose ([^18^F]FDG)-bioradiography as a non-invasive test for estimation of the safety, soundness, and effectiveness of tissues for transplantation, [^18^F]FDG uptake and cell viability or metabolism were investigated using a reconstructed human epidermal model (RHEM). We developed an imaging system, and suitable bioradiographic image acquisition conditions and its effectiveness were investigated. [^18^F]FDG uptake increased in agreement with DNA content as a marker of cell numbers and for histological assessment during cell proliferation and keratinization. [^18^F]FDG uptake was significantly decreased in good agreement with the viability of tissues used with various hazardous chemical treatments. [^18^F]FDG uptake by the tissues was decreased by hypothermia treatment and increased by hypoxia treatment while maintaining cell viability in the tissue. Therefore, [^18^F]FDG-bioradiography can be useful to estimate cell viability or metabolism in this RHEM. This method might be utilized as a non-invasive test for quality evaluation of tissues for transplantation.

## Introduction

Regenerative technologies have already started to cure diseases and injury using transplantable tissues and organs manufactured from embryonic stem cells and induced pluripotent stem (iPS) cells^1^. Sheet-like clusters of cells for regenerative medical uses utilize embryonic stem iPS cell technology, and sheet-like tissue constructs have been developed and begun to be used for the treatment of human diseases^2–5^. Quality control of tissues and organs for transplant is important to confirm their safety and effectiveness for regenerative medicine. Moreover, human embryonic stem cells and iPS cells can also form tumors and they are ethically controversial^4,6^. However, current quality evaluations carried out on tissues and organs for transplant may be insufficient, because comprehensive non-invasive quality evaluation methods have not yet been developed^7,8^. Quality control is primarily carried out through manufacturing control, and product management is secondary. Product management includes inspection for contamination by foreign matter, including cells, and the thickness of the tissue by visual observation. Cell viability, pathological, and microbiological tissue examinations have often been carried out on tissues and organs that have been cultured in parallel with those destined for transplant, because many of the evaluation tests are invasive. For example, the 3-[4,5-dimethylthiazol-2-yl]-2,5-diphenyltetrazolium bromide (MTT) reduction assay is a standard quantitative method that is used to measure cell viability. However, the MTT assay is a destructive method, because the formazan product of the MTT assay accumulates as an insoluble precipitate inside the cells and must be solubilized prior to recoding absorbance. Moreover, longer incubation times result in the accumulation of MTT and increased sensitivity; however, it also causes cytotoxicity due to the detection reagents^9^.

An autoradiographic method termed “bioradiography” has been developed to estimate metabolism and physiological function in living brain slices using positron emitter-labeled compounds for positron emission tomography (PET)^10–12^. This imaging technique can detect dynamic changes in radioactivity in living brain slices by repeated exposure to radioluminographic plates in controlled physiological conditions. We then developed a novel imaging system, “real-time bioradiography”, to acquire bioradiographic images of living tissue in real time using a photon-counting camera and a solid scintillator^13,14^. Tissue 2-[^18^F]fluoro-2-deoxy-D-glucose ([^18^F]FDG) uptake reflects regional glucose uptake based on glycolysis, and it allows the diagnosis of cancer metastasis and differential diagnosis of dementia^15,16^. We considered whether bioradiography and real-time bioradiography using [^18^F]FDG may be suitable for the quality control testing of regenerative medicine products, because [^18^F]FDG was expected to estimate the cell viability and neoplastic transformation in a non-invasive manner.

In order to acquire bioradiographic images of a reconstructed human cultured epidermal model (RHEM) as a cultured epithelium model for grafting using [^18^F]FDG, we developed an imaging system composed of an imaging chamber, radioluminography plate, and culturing incubator, and the optimum image acquisition conditions and the effectiveness of the method were investigated.

## Results

### Performance-based evaluation using ^18^F sources

**Figure 1:**
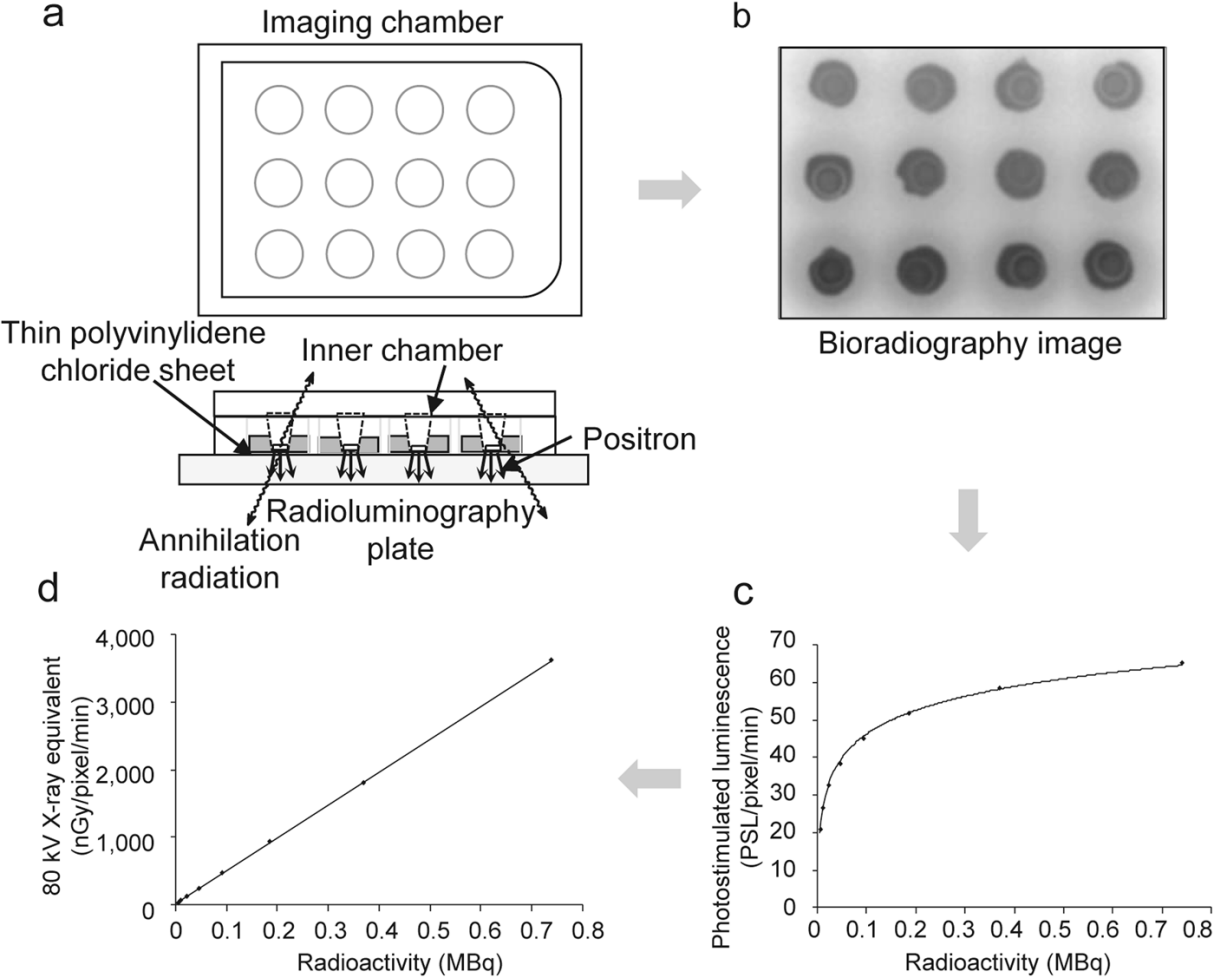
(**a**) Schematic illustration of the imaging chamber, (**b**) bioradiographic image, (**c**) photostimulated luminescence (PSL) intensity obtained using ImageJ analysis expressed as the mean of three samples, and (**d**) converted into 80 kV X-ray equivalent (see Results section for formula).

Autoradiographic images were obtained using bioradiography with [^18^F]FDG, an imaging chamber, and a radioluminography plate (Fig. 1a,b). The imaging data were decay corrected and expressed as “photostimulated luminescence (PSL)/pixel/min”; however, the radioactivity–PSL curves were saturated at high radioactivity concentrations from the ^18^F sources (Fig. 1c). When the values were converted into 80 kV X-ray equivalents using the correction formula: ${\rm{nGy}}=8,770\times 10{}^{(\frac{{\rm{PSL}}-1,535}{1,024}-\mathrm{log}1.25)}$ provided by the manufacturer, a strong positive linear regulation was obtained between ^18^F radioactivity and nGy/pixel/min (Fig. 1d). Therefore, all PSL values in the following experiments were corrected and expressed as nGy/pixel/min using this formula.

### Effect of culture medium [^18^F]FDG concentration on [^18^F]FDG uptake and uptake rate in the RHEM

**Figure 2:**
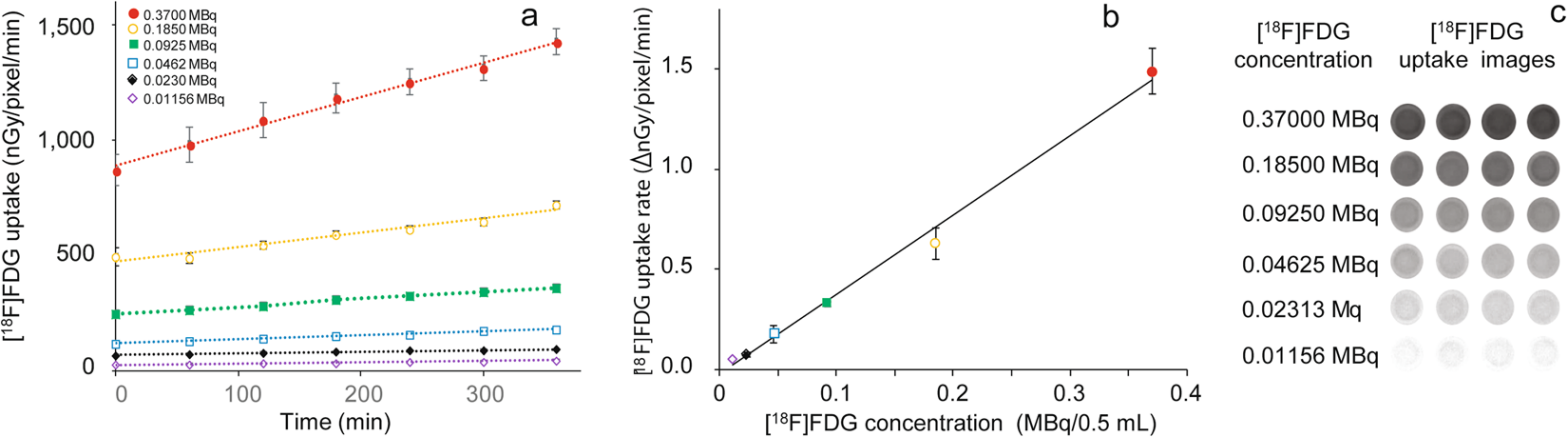
Effect of culture medium [^18^F]FDG concentration on (**a**) [^18^F]FDG uptake and (**b**) [^18^F]FDG uptake rate in the RHEM. The [^18^F]FDG uptake (nGy/pixel/min) and uptake rate (∆nGy/pixel/min) are expressed as mean ± SEM of four tissues. (**c**) [^18^F]FDG uptake images (300–345 min) demonstrated an increase dependent on [^18^F]FDG concentration applied in the medium.

[^18^F]FDG uptake images were obtained every 45 min until 405 min after the start of incubation (Fig. 2a,c). Higher [^18^F]FDG concentrations in the medium were associated with increased [^18^F]FDG uptake (Fig. 2a), and the [^18^F]FDG uptake rate increased linearly with the [^18^F]FDG concentration in the medium up to 0.3 MBq/0.5 mL (Fig. 2b,c).

### Effect of culture medium glucose concentration on [^18^F]FDG uptake and uptake rate in the RHEM

[^18^F]FDG uptake by the tissue decreased in a concentration-dependent manner in accordance with the glucose concentration in the medium up to 20 mM/0.5 mL (Fig. 3a), with the [^18^F]FDG uptake rate decreasing to 89%, 73%, 44%, 39%, and 23% with 1, 2, 5, 10, and 20 mM glucose, respectively (Fig. 3b). [^18^F]FDG uptake images (300–345 min) demonstrated this decrease depending on the glucose concentration in the medium (Fig. 3c).

**Figure 3:**
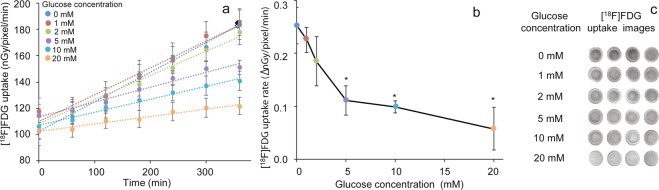
Effect of culture medium glucose concentration on (**a**) [^18^F]FDG uptake and (**b**) [^18^F]FDG uptake rate in the RHEM. The [^18^F]FDG uptake (nGy/pixel/min) and uptake rate (∆nGy/pixel/min) are expressed as mean ± SEM of four tissues. Statistical significance was determined using a non-parametric test (Steel test). Asterisks indicates *p < 0.05. (**c**) ^18^F F]FDG uptake images (300–345 min) demonstrated a decrease depending on glucose concentration applied in the medium.

### Comparison of [^18^F]FDG uptake rate, DNA content, and hematoxylin and eosin (HE) staining histology of the RHEM during cell proliferation and keratinization

**Figure 4:**
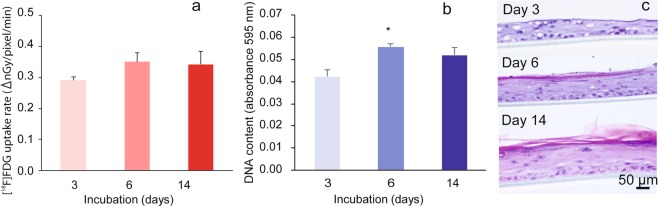
Comparison of (**a**) [^18^F]FDG uptake rate and (**b**) DNA content of the RHEM during cell proliferation and keratinization. The [^18^F]FDG uptake rate (∆ nGy/pixel/min) and DNA contents (absorbance 595 nm) are expressed as mean ± SEM of five tissues. Statistical significance was determined using a non-parametric test (Steel test). An asterisk indicates *p < 0.05. DNA content was determined as a marker of cell quantification. (**c**) Observation of RHEM at days 3, 6, and 14. A cross-section of the tissue was stained with hematoxylin and eosin after formaldehyde fixation.

[^18^F]FDG uptake rate in tissues showed an increase from day 3 to days 6 and 14 after seeding cultured keratinocytes in the cell culture inserts (Fig. 4a). DNA contents, determined using the DPA method as a marker of cell numbers increased with [^18^F]FDG uptake (Fig. 4b). The correlation coefficient (*r*^2^) between the [^18^F]FDG uptake rate and DNA content was 0.797 (p < 0.01). Histological assessment of HE stained tissue sections is shown in Fig. 4c. The nuclei of cells, as a marker for cell numbers, were stained violet, and the results were consistent with [^18^F]FDG uptake and DNA content. Keratinocytes on the cell culture inserts formed a stratified squamous structure similar to cultured epithelial autografts and became thicker with time. The stratum corneum, stained red, appeared from 6 day after the start of culture, and its thickness reached about 30 µm at 14 day.

### Effects of organisation for economic co-operation and development (OECD) test guideline listed chemicals and sodium dodecyl sulfate (SDS) on [^18^F]FDG uptake rate and cell viability of the RHEM

**Figure 5:**
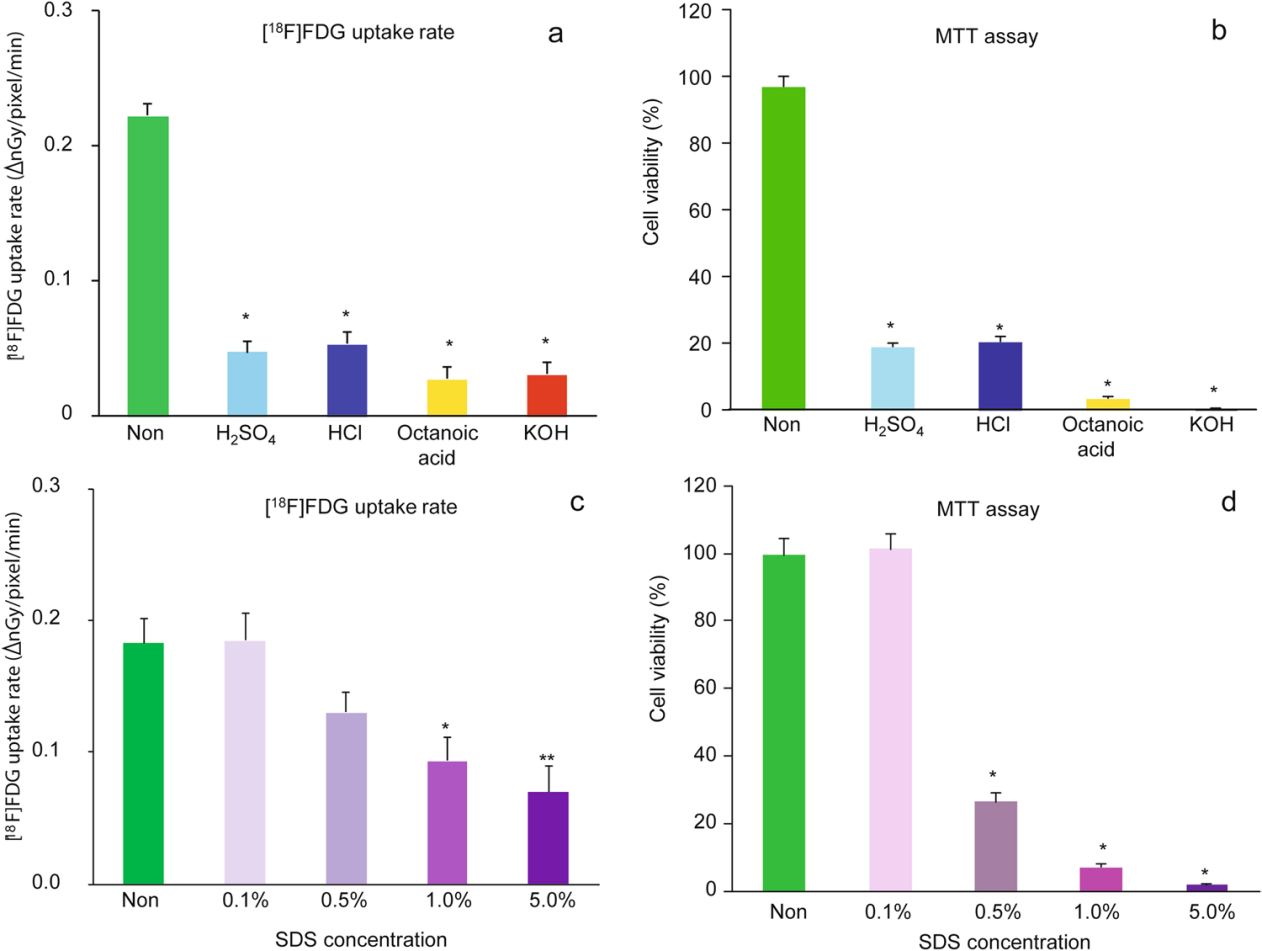
Effect of (**a**,**b**) Organisation for Economic Co-operation and Development (OECD) test guideline listed chemicals and (**c**,**d**) sodium dodecyl sulfate (SDS) on (**a**,**c**) [^18^F]FDG uptake rate and (**b**,**d**) cell viability of the RHEM. Living cells were determined using an MTT assay and expressed as a percentage of the non-treated cells. The [^18^F]FDG uptake rate (∆ nGy/pixel/min) and cell viability (%) are expressed as mean ± SEM of four tissues. Statistical significance was determined using a non-parametric test (Steel test). Asterisks indicates *p < 0.05 or **p < 0.01.

The [^18^F]FDG uptake rate of tissues was significantly reduced by various hazardous chemicals applied to the tissues to 21%, 24%, 12%, and 14% of the control by sulfuric acid, hydrochloric acid, octanoic acid, and potassium hydroxide, respectively (Fig. 5a). The viability of tissues estimated using the MTT assay was also decreased by these chemicals (Fig. 5b). Similarly, the tissue uptake rate of [^18^F]FDG decreased in a concentration-dependent manner with the application of SDS (Fig. 5c). The decrease in the tissue uptake rate of [^18^F]FDG was in good agreement with the viability of the tissue estimated using the MTT assay (Fig. 5d). The correlation coefficient (*r*^2^) between [^18^F]FDG uptake rate and viability was 0.949 (p < 0.01) for the OECD test guideline^17^ listed chemicals and 0.667 (p < 0.01) for SDS.

### Effect of hypothermia and hypoxia on [^18^F]FDG uptake rate and cell viability of the RHEM

**Figure 6:**
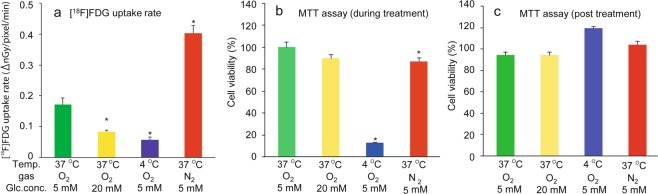
Effect of hypothermia and hypoxia on (**a**) [^18^F]FDG uptake rate and (**b**,**c**) cell viability of the RHEM. Living cells were determined using an MTT assay (**b**) during and (**c**) post treatment, and expressed as a percentage of the non-treated cells. The [^18^F]FDG uptake rate (∆nGy/pixel/min) and cell viability (%) are expressed as mean ± SEM of four tissues. Statistical significance was determined using a non-parametric test (Steel test). Asterisks indicates *p < 0.05 or **p < 0.01.

The effects of hypothermia (4 °C) and hypoxia (95% N_2_/5% CO_2_) on the [^18^F]FDG uptake rate by tissues were also investigated by comparing cell viability using MTT assays. The [^18^F]FDG uptake rate in tissues was significantly decreased to 34% of the control by hypothermia treatment, and the decreased [^18^F]FDG level was approximately equal to the level with 20 mM glucose. On the contrary, the [^18^F]FDG uptake rate in tissues was significantly increased to 235% of the control by hypoxia treatment (Fig. 6a). Cell viability was evaluated during and post hypothermia and hypoxia treatments. When the tissues (at ~405 min) were returned to the control conditions (37 °C, normoxia, and 5 mM glucose) after the hypothermia and hypoxia treatments, the percentage of surviving cells in the tissue was similar to the control value (Fig. 6c). However, the percentage of surviving cells in tissues, as determined using the MTT assay, was significantly decreased to 13% of the control by hypothermia treatment (Fig. 6b).

## Discussion

All previous applied research using bioradiography and real-time bioradiography was carried out on brain tissue slices^10–14^. The thickness of brain tissue slices was about 300 µm, with an estimated depth of 40–50 cells. On the other hand, the thickness of the RHEM in the present study was about ~100 µm, and only a few cells were predicted in this thickness (Fig. 4c). The epidermis is not a significantly functional organ, rather it provides a barrier to infection from environmental pathogens and regulates the amount of water released from the body^18^. Therefore, cell numbers and the metabolic rate in epidermal tissue were predicted to be less than in brain tissue. In order to estimate [^18^F]FDG uptake based on high-quality image acquisition, an imaging chamber for epidermal tissue was developed, and the optimal of image acquisition conditions were investigated. However, no linearity was observed between [^18^F]FDG concentration in the medium and the PSL value in epidermal tissue expressed as “PSL/pixel/min” (Fig. 1c). This characteristic of the radioluminography plate is convenient for identifiability in X-ray contrast images but not for the quantitative measurement of ß^+^-rays from radionuclides in tissue. This non-linear curve was corrected with the 80 kV X-ray equivalent value using a correction formula provided by the manufacturer (Fig. 1d). [^18^F]FDG uptake images were obtained every 45 min for up to 405 min after the start of the incubation (Fig. 2a,c), and a good linear relationship was observed between the [^18^F]FDG concentration (from 0.01156 MBq/0.5 mL to 0.37 MBq/0.5 mL) in the medium and the [^18^F]FDG uptake rate (nGy/pixel/min) in epidermal tissue (Fig. 2b). In terms of reduction in radiation exposure for high-quality image acquisition, 0.04625 MBq/0.5 mL [^18^F]FDG was adapted as a suitable [^18^F]FDG concentration in the medium.

After administration, [^18^F]FDG is transported into the cell *via* the glucose transporter (GLT) and is phosphorylated by hexokinase and trapped intracellularly^15^. Therefore, trapped ^18^F radioactivity in tissues or cells reflects the activity of glucose metabolism based on the GLT and hexokinase. All organs as well as skin possess a glycolysis pathway in cells as part of their metabolism. Skin is a unique organ, because epidermal cells in the skin are not directly vascularized, and glucose and oxygen are transported from the dermis by simple diffusion^18^. GLT1 and GLT2 proteins are expressed in the stratum corneum of epidermal cells, but GLT1 expression becomes more restricted to the stratum basale with development. Although GLT2 is found mainly in the stratum spinosum and granulosum, it is not localized in the stratum basale^19^. GLT1 expression in the basal layer of the epidermis is modulated by keratinocyte differentiation^20^. GLT1 and the glutamate receptor are colocalized in basal layer keratinocytes, and a role for glutamate in the control of epidermal renewal has been suggested^21^. In a pathological condition, GLT1 is strongly expressed in the epidermis of involved psoriatic lesions and during wound healing^22^. In the present study, [^18^F]FDG uptake and the uptake rate in tissue decreased in a concentration-dependent manner by the addition of glucose to the medium up to 20 mM/0.5 mL (Fig. 3). This result suggested that glucose-specific processes, GLT, and hexokinase may be involved in [^18^F]FDG uptake in this RHEM. Lower glucose concentration in the medium contributed to higher [^18^F]FDG uptake and high-quality image acquisition in the tissue (Fig. 3). Consequently, 5 mM glucose was determined as a suitable concentration in the medium for the following experiments, with consideration of the physiological glucose concentration in serum. Comparison of [^18^F]FDG uptake rate, DNA content, and HE staining histology of the RHEM during cell proliferation and keratinization is shown in Fig. 4. During cell proliferation and keratinization, the stratum corneum, stained in red, appeared from 6 days after the start of culturing, and the thickness reached about 30 µm at 14 day. Keratinocytes on the cell culture inserts formed a stratified squamous structure similar to a cultured epithelial graft (Fig. 4c). The [^18^F]FDG uptake rate in tissues showed a slight increase from day 3 to days 6 and 14 after seeding the cultured keratinocytes on the cell culture insert (Fig. 4a). Histological assessment of HE-stained tissue sections is shown in Fig. 4c, with the nuclei of cells stained violet. The histological assessment (Fig. 4c) indicated that a layer of keratinocytes as basal cells formed on the cell culture inserts and then several layers of cells formed on the basal cells. DNA contents determined using the DPA method as a marker for cell numbers increased similarly with [^18^F]FDG uptake (Fig. 4b). The results of histological assessment and DNA content in the tissues were consistent with [^18^F]FDG uptake. This suggested that the tissue uptake of [^18^F]FDG can be an indicator of the number of cells per unit area. However, the stratum corneum does not contribute to the uptake of [^18^F]FDG because it is the outermost layer of the epidermis and consists of dead cells. The standardized uptake value (SUV) is a popular semi-quantitative value for the evaluation of PET as well as single photon emission tomography images for comparison between subjects^23^. SUV is calculated using the ratio of the image-derived radioactivity concentration and the whole body concentration of the injected radioactive compound. In the present study, the [^18^F]FDG concentration in medium was calculated from the added dose of [^18^F]FDG and the volume of the medium. However, the image-derived radioactivity concentration per volume or thickness is not easily obtained. It will be possible to calculate the value using data on the thickness distribution of tissues, which can be estimated from the radiation and optical^24,25^ attenuation coefficient map. The radiation attenuation coefficient can be obtained from the uptake value difference in the autoradiographic images between no tissue (background) and no uptake tissue (at time zero) similar to the attenuation correction using transmission data in PET^26^ (see Supplementary Fig. S1).

The Draize test is an acute toxicity test, devised by Draize and Spines, which is used for the initial testing of cosmetics and then irritant chemicals to the eye or skin of a conscious animal^27^. The Draize rabbit eye and skin irritation test was set as a gold standard by the OECD. However, it has been criticized with respect to animal welfare due to it being an invasive and cruel procedure. To replace the Draize test, various alternatives have been developed, such as 3D reconstructed human cornea-like epithelium and epidermis models^28,29^. The OECD 2015 guidelines present test guideline for *in vitro* procedures that may be used for the hazard identification of irritant chemicals. The 3D RHEM used in the present study is one of the models that the OECD 2015 adapted for *in vitro* procedures^17^. This test guideline also recommended the MTT assay to assess cell viability in tissues and to quantify the hazard of irritant chemicals. In the present study, we examined the effects on [^18^F]FDG uptake of various hazardous chemicals mentioned in the guideline, and we investigated in tissues by evaluating cell viability using MTT assays (Fig. 5). [^18^F]FDG uptake was significantly decreased by various hazardous chemicals (sulfuric acid, hydrochloric acid, octanoic acid, and potassium hydroxide) applied to the tissues (Fig. 5a). Similarly, the tissue uptake of [^18^F]FDG decreased in a concentration-dependent manner by addition of the SDS (Fig. 5c). We had also examined the effect of heat treatment on [^18^F]FDG uptake rate and cell viability in the RHEM. However, [^18^F]FDG uptake was not decreased by heat treatment (at least 60 °C for 5 min) in agreement with the cell viability of tissue estimated using the MTT assay (see Supplementary Fig. S2). The [^18^F]FDG uptake rate and the percent viability in control and heat-treated samples were 0.249 ± 0.036 and 0.212 ± 0.024, and 101.2 ± 2.4% and 106.0 ± 3.4%, respectively. In a previous study, we demonstrated a significant decrease in labeled oxygen uptake by heat treatment of brain slices^30^. The 3D cultured RHEM might be more resistant to heat treatment than brain slices. Tissue [^18^F]FDG uptake was in good agreement with that of the viability of tissues estimated using the MTT assay (Fig. 5b,d). These results suggested that tissue uptake of [^18^F]FDG reflects cell viability in the tissue. In order to investigate the relationship between [^18^F]FDG uptake and energy metabolism in tissues, the effects of hypothermia (4 °C) and hypoxia (95% N_2_/5% CO_2_) on [^18^F]FDG uptake were also examined by comparing cell viability using the MTT assay method, as in Fig. 6. The [^18^F]FDG uptake rate in tissue was significantly decreased to 34% of the control by hypothermia treatment, and the decreased [^18^F]FDG level was approximately equal to the level with 20 mM glucose (Fig. 6a), and the survival percentage of cells in tissues with MTT assays was significantly decreased to 13% of the control by hypothermia treatment (Fig. 6b). However, when these samples were returned to the control conditions, the survival percentage of cells in the tissue was not different to the control value (Fig. 6c). From these results, we can make an interpretation that the MTT assay showed a false decrease in cell viability in tissues after hypothermia treatment, because the MTT assay was used to indicate the reduction in MTT to purple formazan in living mitochondria. On the contrary, the [^18^F]FDG uptake rate in the tissue was significantly increased to 235% of the control by hypoxia treatment (Fig. 6a). Cell viability was evaluated using MTT assays maintained during and post hypoxia treatment. (Fig. 6b,c). In aerobic conditions, with 29.85 ATP being generated from one molecule of glucose by glycolysis and oxidative phosphorylation, whereas it produces just 2 ATP in anaerobic conditions by glycolysis^31^. Inhibition of oxidative phosphorylation during hypoxia treatment may cause enhanced glycolysis, and it may be involved in the enhancement of [^18^F]FDG uptake into epidermal tissues. A similar phenomenon has been demonstrated in our previous report with brain tissue slices^12,13^. These results suggested that the tissue uptake of [^18^F]FDG reflects the tissue viability and metabolic activity.

**Figure 7:**
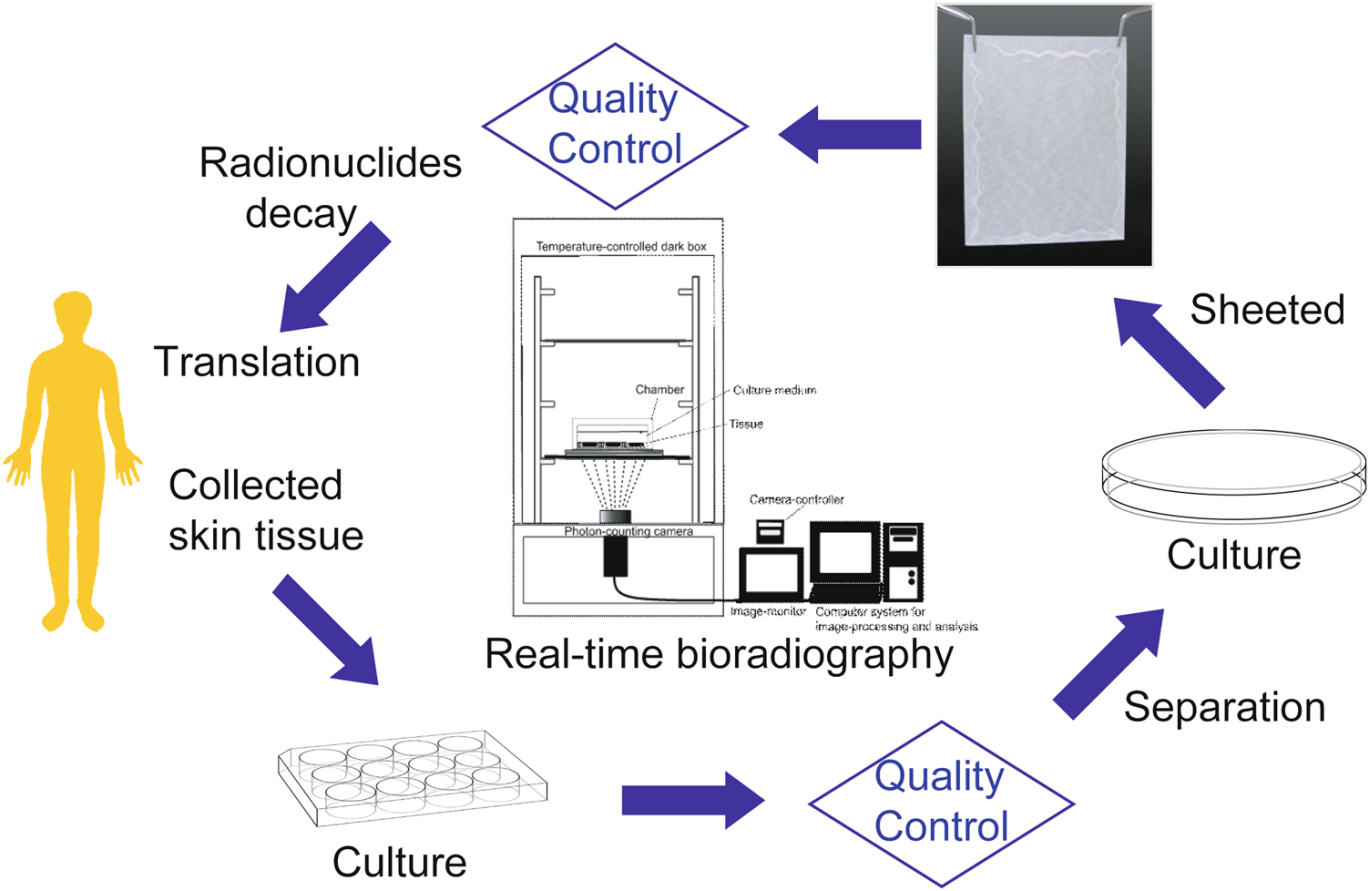
Proposal for quality control in regenerative medicine products using real-time bioradiography and bioradiography.

Thus, [^18^F]FDG-bioradiography enabled the non-invasive and dynamical evaluation of cell viability and metabolic activity in cultured tissues, and this method is expected to be used in the quality control of regenerative medicine products, and in research and development of regenerative medicine (Fig. 7). In addition, [^18^F]FDG-bioradiography may be useful to identify neoplastic transformation non-invasively in regenerative medicine products. We also considered that [^18^F]FDG-bioradiography has other advantages and disadvantages as a quality evaluation for transplant tissues and organs. Radiolabeled probes used to be the most common type, but recently these have been replaced by non-radioactive labels, such as fluorescent and chemiluminescent labels, because of safety considerations as well as the cost and difficulty in the disposal of radioactive waste products. Compared with radioactive labels, the use of non-radioactive labels has several advantages. However, chemical contamination in tissues and organs for transplant is a concern based on the retained micromolar order of non-radioactive labels. The tracer (picomolar) order of radioactive labels is so small that it hardly counts as chemical contamination. Therefore, [^18^F]FDG has no toxic effect in trace amounts. In addition, radiolabeled probes are the most sensitive. Concern over the safety and economic and environmental aspects of radioactive waste disposal have been key factors in radioactive label use. Radionuclides for PET have an extremely short half-life. For example, almost all ^18^F 0.0425 MBq (4.40 × 10^8^ atoms) radioactivity decays within 24 hours, reaching the last nuclide at 52.5 hours (see Supplementary Fig. S3). Another concern is the risk of a recipient receiving tissue with a history of radiation exposure from ^18^F. The risk was roughly estimated as follows. The absorbed dose of positrons and annihilation photons from [^18^F]FDG in the medium and that entered the tissue was estimated as 6 mGy (mJ/kg tissue) tentatively using the MIRD^32,33^. If cultured epithelial grafts equivalent to the RHEM with a history of radiation exposure where transplant across the entire surface of burns to a patient’s body, the effective dose of radiation to the patient was estimated to be 0.06 mSv using a radiation weighting factor of 1 and a tissue weighting factor of 0.01^34^. This effective dose was well below the dose limit for the general public (1 mSv/year) prescribed by the International Commission on Radiological Protection regulation (ICRP Pub. 103)^34^. Stricter and more detailed effective dose estimation will require further investigation. Accuracy and precision are important concepts to compare the reliability of different methods for measuring a quantity. The precision of the measurement can compare between [^18^F]FDG-bioradiography and MTT assay. The coefficient of variation (CV) is the ratio of the standard deviation or SEM to the mean. It allows comparison between the distribution of values whose scales of measurement are not comparable. The calculated CV ((SEM/mean) × 100%) in non-treated tissues for [^18^F]FDG-bioradiography and the MTT assay was 5–13% and 3–5%, respectively. The higher CV for the [^18^F]FDG-bioradiography than the MTT assay indicated the lower measurement precision in former than the latter. [^18^F]FDG has established safety as a radiopharmaceutical and is supplied commercially by radiopharmaceutical companies. The price of [^18^F]FDG is higher than that of the MTT regent, whereas the quality control in regenerative medicine products using [^18^F]FDG-bioradiography is more valuable than that of the price, because it may be able to estimate cell viability in the products for transplant. 3D cultured human epidermal and corneal models have been developed to study the irritation potential induced by drugs, pesticides, chemicals, or cosmetics on the skin or eyes without animal studies^35–38^. These models are employed in the OECD guideline for the testing of chemicals^17^. As described above, the MTT assay as a cell viability measurement in the OECD 2015 test guideline is invasive. We consider that [^18^F]FDG-bioradiography is useful as a replacement for *in vitro* skin corrosion tests based on the OECD guideline.

In conclusion, [^18^F]FDG-bioradiography is expected to be applicable as a non-invasive, dynamical evaluation method of tissues for transplantation and a replacement for *in vitro* tests based on the OECD.

## Methods

### Performance-based evaluation of the equipment using an ^18^F source

Multiwell cell culture plates with 12 covered wells (FALCON Multiwell 353043; Corning, NY, USA) were used to prepare the imaging chamber. A 12-mm diameter hole was drilled in the bottom of each well. A thin polyvinylidene chloride sheet (30 µm thick) was glued tightly to the border of the bottom of the well. [^18^F]FDG (111 MBq/1.75 mL 0.9% NaCl; FUJIFILM Toyama Chemical Co., Ltd., Tokyo, Japan) was diluted with distilled water to eight concentrations of [^18^F]FDG solution (5.781 × 10^−3^ MBq/0.5 mL, 0.0115625 MBq/0.5 mL, 0.023125 MBq/0.5 mL, 0.04625 MBq/0.5 mL, 0.0925 MBq/0.5 mL, 0.185 MBq/0.5 mL, 0.37 MBq/0.5 mL, and 0.74 MBq/0.5 mL). Each concentration of [^18^F]FDG solution (n = 3) was applied to the imaging chamber and placed on the radioluminography plate (CR palate; REGIUS RP-4S 8 × 10 cm; KONICA MINOLTA JAPAN, INC., Tokyo, Japan) without a cassette (REGIUS RP4S110; KONICA MINOLTA JAPAN, INC., Tokyo, Japan) for 45 min in a CO_2_ incubator (Direct Heat CO_2_ Incubator SCA-30D; ASTEC CO., Ltd., Fukuoka, Japan). Autoradiographic images recorded on the plate were read using a REGIUS MODEL 170 (KONICA MINOLTA JAPAN, INC., Tokyo, Japan) and analyzed using ImageJ^39^. A region of interest (ROI) was placed on whole image of each ^18^F source. For quantitative analysis, the values were decay corrected. Radioactivity was expressed as “PSL/pixel/min”.

### Effect of culture medium [^18^F]FDG concentration on [^18^F]FDG uptake and uptake rate in a RHEM

RHEM (LabCyte EPI-MODEL 24 Day 6; Japan Tissue Engineering Co., Ltd., Gamagori, Japan) tissues were preincubated on an inert filter substrate (FALCON Cell Culture Insert for 24-well format 353095; Corning, NY, USA) at an air–liquid interface in a 24-well cell culture plate (FALCON Multiwell 353047; Corning, NY, USA) containing culture medium with fetal bovine serum (cat. No. 402250). The tissues were cultured in a CO_2_ incubator (95% air/5% CO_2_) at 37 °C with saturated humidity for 60 min. Then, the tissues were transferred to the imaging chamber with 5 mM glucose and different concentrations of [^18^F]FDG (0.0115625 MBq, 0.023125 MBq, 0.04625 MBq, 0.0925 MBq, 0.185 MBq, and 0.37 MBq) in 0.5 mL of Dulbecco’s modified Eagle medium (DMEM) medium and incubated in the CO_2_ incubator. DMEM medium containing 5 mM glucose was prepared from DMEM with L-glutamine and without D-glucose and sodium pyruvate (11966-025), and with 4.5 g/L D-glucose glucose and L-glutamine and without sodium pyruvate (11965-092). Two-dimensional images of radioactivity in the tissues were recorded on a CR plate through a thin polyvinylidene chloride sheet (30 μm thick) placed at the bottom of the chamber. Autoradiographic images recorded on the plate were read using a REGIUS MODEL 170. An ROI was placed on the whole image of the tissue and analyzed using ImageJ. [^18^F]FDG uptake was expressed as “PSL/pixel/min”. Dynamic changes in radioactivity in the tissues were measured by exposing the plate for 45 min and exchanging it every 60 min for 405 min. For quantitative analysis, the values were decay corrected and converted into 80 kV X-ray equivalents using the correction formula provided by the manufacturer (KONICA MINOLTA JAPAN, INC.) and were expressed as “nGy/pixel/min”. Uptake rate in [^18^F]FDG was obtained by plotting the [^18^F]FDG uptake *vs*. incubation time and expressed as “∆nGy/pixel/min”. Data were expressed as mean ± SEM of four tissues.

### Effect of culture medium glucose concentration on [^18^F]FDG uptake and uptake rate in the RHEM

Preincubated tissues (LabCyte EPI-MODEL 24 Day 6) as described above were transferred to the imaging chamber with 0.04625 MBq [^18^F]FDG and different concentrations of glucose (1, 2, 5, 10, and 20 mM) in 0.5 mL DMEM medium, and were incubated at 37 °C in the CO_2_ incubator. Different concentrations of glucose in DMEM medium were prepared by adjusting the mixing ratio of from 0 g/mL D-glucose to 4.5 g/L D-glucose. Dynamic changes in radioactivity in the tissues were measured by exposing the plate, and [^18^F]FDG uptake and uptake rate were obtained as mentioned above. Data were expressed as mean ± SEM of four tissues.

### Comparison of [^18^F]FDG uptake rate, DNA content, and HE stained histology of the RHEM during cell proliferation and keratinization

The RHEM (LabCyte EPI-KIT 401810) was purchased from Japan Tissue Engineering Co., Ltd. (Gamagori, Japan). Three-dimensional cultures of human epithelial cells grown based on the method described by Hanada *et al*.^40^. Cryopreserved keratinocytes grown in the presence of irradiated 3T3 cells were thawed and suspended in the culture medium. Cell aliquots were seeded into cell culture inserts placed in 24-well Multiwell cell culture plates (FALCON Multiwell 353047; Corning, NY, USA) included in the manufacturer’s kit, where each well was filled with 1.5 ml of culture medium containing fetal bovine serum (cat. No. 402250) and incubated in a CO_2_ incubator. The culture medium in the well was changed every 3 days. Keratinocytes seeded in the cell culture insert were submerged when the medium was present in the insert. Upon removing the medium in the insert, the keratinocytes were exposed to air on the upper side, resulting in a culture at an air-liquid interface. After 3, 6, and 14 days, epidermal tissues on an inert filter substrate were rinsed with DMEM medium containing 5 mM glucose, then transferred to 0.5 mL of DMEM medium containing 0.04625 MBq [^18^F]FDG and 5 mM glucose in the imaging chamber, and were incubated in the CO_2_ incubator. Dynamic changes in radioactivity in the tissues were measured by exposing the plate, and [^18^F]FDG uptake and the uptake rate were obtained as mentioned above. After assays for DNA content, epidermal tissues on the inert filter substrate were rinsed twice with phosphate-buffered saline (PBS), clipped, and transferred to 1 mL of reaction solution containing 0.6 g diphenylamine (DPA)/15 mL acetic acid (4% w/v):20% acetaldehyde (0.16% v/v)/perchloric acid (20% v/v) = 5:3 (v/v) in a Teflon sealed glass vial at 37 °C for 24 hours based on the method described by Pharm *et al*.^41^. The resulting blue color in the reacted solution (0.25 mL) was measured as the absorbance value at 595 nm in a 96-well microplate using microplate reader (Multiskan GO 51119350; Thermo Fisher Scientific, MA, USA). All data were expressed as mean ± SEM of five tissues. For histological assessment, three of the epidermal tissues on the cell culture insert were removed at 3, 6, and 14 days and fixed 4% paraformaldehyde in 0.1 M phosphate buffer (pH 7.4) and embedded in paraffin. Tissue sections (thickness; 5 µm) were stained with HE.

### Effect of OECD test guideline listed chemicals and SDS on [^18^F]FDG uptake rate and cell viability of the RHEM

OECD test guideline listed chemicals (10% w/v H_2_SO_4_, 14.4% w/v HCl, octanoic acid, and 10% w/v KOH) in 50 µL of distilled water and different concentrations of SDS (0.1%, 0.5%, 1%, and 5% v/v) in 50 µL of PBS applied to the tissues (LabCyte EPI-MODEL 24 Day 6) on cell culture inserts and incubated continuously for 15 min in the CO_2_ incubator. As a negative control, 50 µL of water or PBS was applied to the tissues. Tissues on the cell culture inserts were rinsed with 0.5 mL of PBS three times and transferred to the imaging chamber containing 0.04625 MBq [^18^F]FDG and 5 mM glucose in 0.5 mL DMEM medium, and were incubated in the CO_2_ incubator. Dynamic changes in radioactivity in the tissues were measured by exposing the plate, and [^18^F]FDG uptake rates were obtained as mentioned above. Data were expressed as mean ± SEM of four tissues. Then, the tissues were transferred to a 24-well plate containing 0.5 mL of freshly prepared MTT medium (1 mg/mL; FUJIFILM Wako Pure Chemical Corporation, Osaka, Japan) to estimate the viability of tissues using the MTT assay, as found previously^9^. Tissues were incubated for 3 hours in the CO_2_ incubator. The purple formazan product was completely solubilized in vials containing 200 µL of isopropanol through an overnight extraction in a refrigerator. Subsequently, 150 µL of extract was transferred to a 96-well microplate, and absorbance values were measured at 570 nm and at 650 nm as a reference using a microplate reader (Multiskan GO 51119350). Tissue viability was calculated as a percentage of the negative control. All data were expressed as mean ± SEM for four tissues.

### Effect of hypothermia and hypoxia on [^18^F]FDG uptake rate and cell viability in the RHEM

For this experiment, a flow imaging chamber was developed in which an imaging chamber was modified to allow gas exchange and humidification in a temperature-controlled incubator (COOL INCUBATOR 41-0440; SANSYO Co., LTD., Tokyo, Japan). A gas inlet was created in the center of the cover, and the vent was open to the atmosphere outside the chamber, allowing continuous gas flow through the chamber. Preincubated tissues (LabCyte EPI-MODEL 24 Day 6) as described above were transferred to the flow imaging chamber containing 0.04625 MBq [^18^F]FDG, and 5 mM or 20 mM glucose in 0.5 mL DMEM medium and incubated at 37 °C or 4 °C in the incubator. Normoxic (75% N_2_/20% O_2_/5% CO_2_) or hypoxic gas (95% N_2_/5% CO_2_) was humidified and supplied into the imaging chamber *via* the gas inlet at a flow rate of 50 mL/min. Dynamic changes in radioactivity in the tissues were measured by exposing the plate, and the [^18^F]FDG uptake rate was obtained as mentioned above. Data were expressed as mean ± SEM of four tissues. After the treatments, the tissues were transferred to 24-well plates containing 0.5 mL of freshly prepared MTT medium to estimate the viability of tissues using the MTT assay. To estimate the viability of tissues during treatment, MTT assays were also carried out for another set of tissues. All data were expressed as mean ± SEM of four tissues.

### Statistics

All data were expressed as mean ± standard error of the mean (SEM), and all experiments were repeated at least twice. Statistical significance was determined using a non-parametric test (Steel test). The relationships between [^18^F]FDG uptake and DNA content or MTT value was evaluated using Spearman’s rank correlation coefficient. A value of p < 0.05 was considered statistically significant.

### Compliance with ethical standards

All procedures performed in the study were in accordance with the ethical standards of the institutional and/or national research committee and with the 1964 Helsinki Declaration and its later amendments or comparable ethical standards.

## Supplementary information

Supplementary Figures
