# Supplementary material for: Evaluation of cell viability and metabolic activity of a 3D cultured human epidermal model using a dynamic autoradiographic technique with a PET radiopharmaceutical
Source: Sci Rep. 2019 Jul 23;9:10685. doi: 10.1038/s41598-019-47153-0 (PMC6650402; doi:10.1038/s41598-019-47153-0)
Supplement: Supplementary file 1 — Supplementary Figures [file 41598_2019_47153_MOESM1_ESM.pdf]

Evaluation of cell viability and metabolic activity of a 3D cultured human epidermal model using a dynamic autoradiographic technique with a PET radiopharmaceutical

Toru Sasaki[1, 2], Junya Tamaki[1], Kentaro Nishizawa[1], Takahiro Kojima[1], Ryoich Tanaka[1], Ryotaro Moriya[1], Haruyo Sasaki[1, 2], Hiroko Maruyama[2]

[1]Department of Medical Engineering and Technology, Kitasato University School of Allied of Health Sciences, 1-15-1 Kitasato, Sagamihara, Kanagawa 252-0373, Japan. [2]Research Facility of Regenerative Medicine and Cell Design, Kitasato University School of Allied of Health Sciences, 1-15-1 Kitasato, Sagamihara, Kanagawa 252-0373, Japan.

Correspondence to: Toru Sasaki; Department of Medical Engineering and Technology, Kitasato University School of Allied of Health Sciences, 1-15-1 Kitasato, Sagamihara, Kanagawa 252-0373, Japan
Tel.: +81 42 778 8157; Fax: +81 42 778 9628
E-mail address: tsasaki@kitasato-u.ac.jp

FIGURES

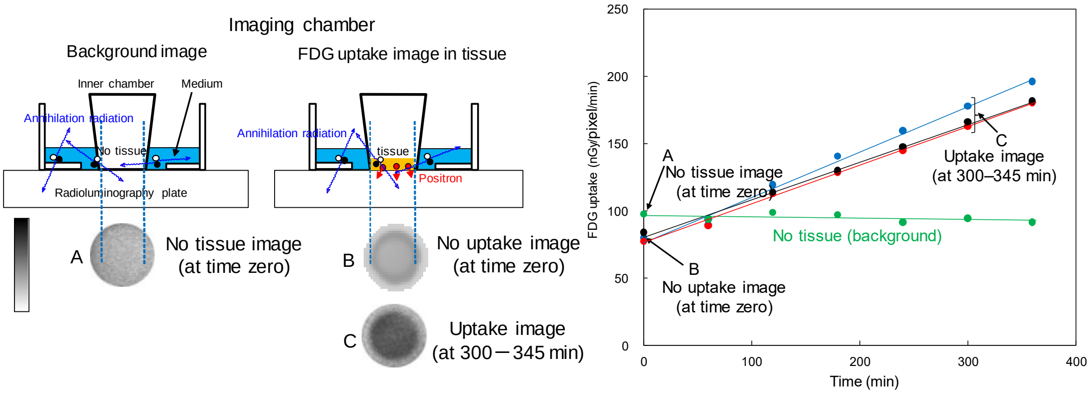

Figure S1. The thickness distribution in tissues can be estimated from the radiation attenuation coefficient map. The radiation attenuation coefficient is calculated as the uptake ratio of the (B) no uptake image (at time zero) and (A) no tissue image (background) similar to the attenuation correction using the transmission data in PET.

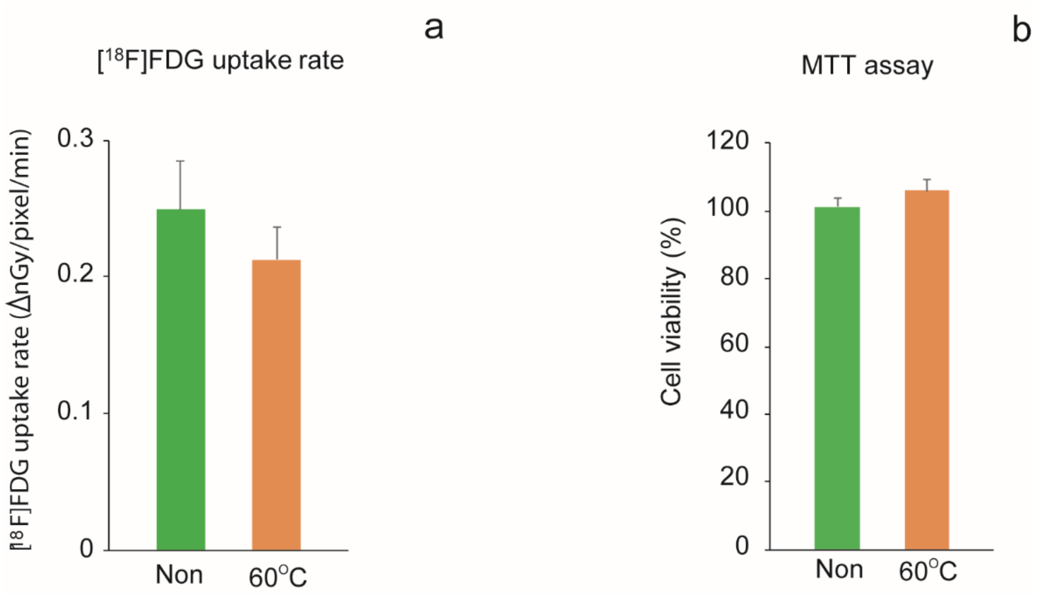

Figure S2. Effect of heat treatment (60°C for 5 min) on (a) [^{18}F]FDG uptake rate and (b) cell viability of the RHEM. Living cells were determined using an MTT assay and expressed as a percentage of non-treated cells. The [^{18}F]FDG uptake rate (ΔnGy/pixel/min) and cell viability (%) are expressed as mean ± SEM of four tissues.

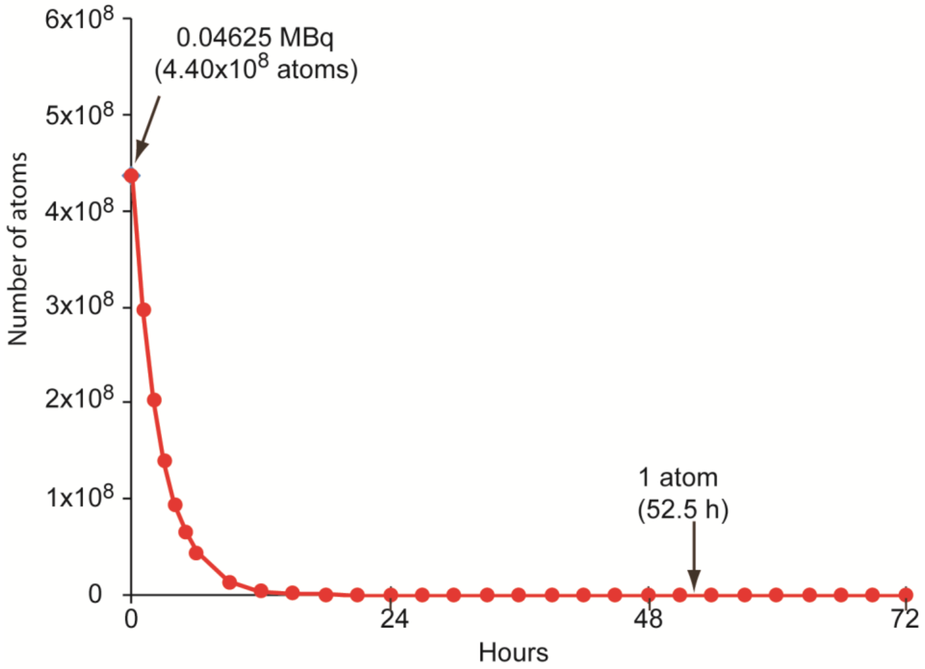

Figure S3. Decay curve of ^{18}F radioactivity. The radioactivity (0.04625 MBq; number of fluorine atoms 4.40×10^8) at time zero decayed to one atom by 52.5 hours.
